# Internet-based self-help treatment for depression in multiple sclerosis: study protocol of a randomized controlled trial

**DOI:** 10.1186/1471-244X-12-137

**Published:** 2012-09-11

**Authors:** Rosa E Boeschoten, Joost Dekker, Bernard MJ Uitdehaag, Chris H Polman, Emma H Collette, Pim Cuijpers, Aartjan TF Beekman, Patricia van Oppen

**Affiliations:** 1 Department of Psychiatry, VU University Medical Center Academic Anxiety Outpatient Clinic and GGZinGeest, A. J. Ernststraat 1187, Amsterdam, HL, 1081, The Netherlands; 2 EMGO Institute for Mental Health and Care Research, VU University, Amsterdam, The Netherlands; 3 Department of Neurology, VU University Medical Center, Amsterdam, The Netherlands; 4 Department of Epidemiology and Biostatistics, VU University Medical Center, Amsterdam, The Netherlands; 5 Department of Medical Psychology, VU University Medical Center, Amsterdam, The Netherlands; 6 Department of Clinical Psychology, VU University, Amsterdam, The Netherlands

**Keywords:** Depression, Multiple sclerosis, Internet, Treatment, Randomized controlled trial

## Abstract

**Background:**

Depression in MS patients is frequent but often not treated adequately. An important underlying factor may be physical limitations that preclude face-to-face contact. Internet-based treatment showed to be effective for depressive symptoms in general and could thus be a promising tool for treatment in MS.

**Methods/design:**

Here, we present a study protocol to investigate the effectiveness of a 5 week Internet-based self-help problem solving treatment (PST) for depressive symptoms in MS patients in a randomized controlled trial. We aim to include 166 MS patients with moderate to severe depressive symptoms who will be randomly assigned to an Internet-based intervention (with or without supportive text-messages) or waiting list control group. The primary outcome is the change in depressive symptoms defined by a change in the sum score on the Beck Depression Inventory (BDI-II). Secondary outcomes will include measures of anxiety, fatigue, cognitive functioning, physical and psychological impact of MS, quality of life, problem solving skills, social support, mastery, satisfaction and compliance rate. Assessments will take place at baseline (T0), within a week after the intervention (T1), at four months (T2) and at ten months follow-up (T3: only the intervention group). The control group will be measured at the same moments in time. Analysis will be based on the intention-to-treat principle.

**Discussion:**

If shown to be effective, Internet-based PST will offer new possibilities to reach and treat MS patients with depressive symptoms and to improve the quality of care.

**Trial Registration:**

The Dutch Cochrane Center, NTR2772

## Background

Multiple Sclerosis (MS) is a chronic and progressive inflammatory autoimmune disorder of the central nervous system. MS is a relatively common disease -affecting approximately 1–2 per 1000 individuals- and mainly commences in early to middle adult life. MS patients suffer from a variety of neurological symptoms such as fatigue, poor balance, impaired speech, bladder and bowel dysfunction, weakness, pain, spasms, cognitive deficits [1,2].

Lifetime risks for depressive disorders in MS patients are high (~50%) [3]. Findings thus far indicate that depression seems more severe and common in MS compared with healthy people. However, due to overlap of MS symptomatology with neurovegetative depressive symptoms such as fatigue, sleeping problems and cognitive impairment, estimated depression prevalence in MS might be overrated. It also remains unclear to what degree depressive symptoms are a neurological consequence of the disease or a psychological reaction to the presence of a chronic medical condition with an uncertain and unpredictable course [4]. Some studies that compared depression in MS with other neurologic conditions and chronic diseases, suggest that depression in MS can be partly attributed to the additional neurological impact of MS [4-6].

Irrespective of its unclear etiology, depression in MS patients causes significant suffering and disability. Depression is associated with fatigue, cognitive impairment, and poorer social support [7]. Moreover, it is related to lower quality of life [8], increased risk of suicide [9] and may adversely affect health status via effects on the immune system or indirectly by influencing behavior that affects risk of MS exacerbation [10,11].

However, depression in MS patients often stays undiagnosed [12]. There may be several explanations for the high level of unrecognized depression in MS patients. It is suggested that MS patients are not actively screened and diagnosed by their clinician on depression. In addition, patients could feel resistance to disclose their emotional problems or perceive them as an unsolvable component of the disease, therefore leaving them unmentioned [3]. Besides, as a result of overlapping symptomatology, confounded symptoms may be entirely attributed to MS when in fact a portion of those symptoms are attributable to depression. However, depression does not seem to remit spontaneously and may even worsen over time if not treated [11].

MS patients seem to respond well to psychotherapeutic and / or medical treatment for depression [13,14]. Depression in MS patients seems to be related to poor problem solving skills [7], and learning various coping strategies has been found to reduce symptoms of depression in MS patients [14]. Research shows that Cognitive Behaviour Therapy (CBT) with a focus on developing sufficient coping skills is preferred to insight-oriented or supportive group therapy [11,14,15]. Problem Solving Therapy (PST) is a form of CBT and assumes that depressive symptoms can be caused by (practical) problems people face in their daily lives combined with poor problem-solving skills. PST focuses on enabling people to solve problems by teaching them more adequate problem-solving skills and helping to accept those problems that cannot be changed [16,17]. PST in particular seems a favourable treatment for people with depressive symptoms and a somatic disease [18]. Nevertheless, the literature on PST for depression in MS patients is limited [19].

Given the evidence for its responsiveness to treatment, it is remarkable that depression in MS patients is so infrequently treated [3,13]. Apart from general obstacles as lack of time, no self-perceived need for care or stigma associated with treatment, MS patients may have disease-related barriers such as transportation problems, physical immobility, fatigue and exacerbations of the disease that might interfere with having face-to-face treatment [14]. Consequently, medication consults or face-to-face psychotherapy may not be feasible forms of treatment for some MS patients [20]. Therefore, alternative treatment delivery should be considered to increase access to mental health care. Telephone administered CBT has previously shown to be more effective in reducing depressive symptoms in MS patients compared to MS patients receiving supportive emotion-focused therapy [21] or no mental healthcare at all [22]. Recently, the Internet has grown as an important tool for delivering mental health interventions [23]; Internet-based CBT or PST appear to be as effective as face-to-face therapy [24] and a successful method of treatment for depression in general [24,25]. Internet-based treatment is easily accessible, cost-effective and can reach a large number of people with functional impairments due to physical health problems which makes it an attractive treatment for the MS population.

However, there are few publications on Internet-based treatment for depressed MS patients. Recently, a multicenter trial was suggested, based on a pilot study that explored Internet-based CBT for the treatment of depression [26]. Qualitative data from this research group showed that existing Internet-based CBT packages might not just be appropriate for MS patients due to MS related physical and cognitive impairments, inappropriate content, social isolation and problems with computer use [27]. However, data from our recent pilot study on Internet-based treatment for depression in MS showed evidence that Internet-based self-help PST treatment [28], adjusted to MS patients, can be a feasible treatment [29]. Patients reported satisfaction with the intervention which reduced depressive symptoms, especially in those MS patients who completed the intervention. These findings encourage us to examine the effectiveness of this Internet-based self-help course for treatment of depressive symptoms in MS in a randomized controlled trial (RCT) with longer follow-up. With an easily accessible Internet-based self-help intervention we hope to improve the level of care and treat a group of MS patients with depressive symptoms who could experience disease-related barriers to participate in face-to-face counselling.

Unfortunately, dropout percentages from Internet-based interventions can be high and do not get enough attention [30]. Also in our pilot study, almost half of the patients did not complete the intervention. Ways to increase compliance rates should therefore be considered. However, specific components that are critical in improving compliance are difficult to identify and experimental manipulation of factors likely to increase compliance in e-health trials are scarce [31]. A meta-analysis suggests that use of mobile phones and text-messaging could improve healthcare outcomes and the processes of care [32] and a RCT showed that telephone reminders increased the frequency of visits to the site of a self-help Internet program for depressive symptoms compared to no reminders [33]. Recently, Mohr and colleges [34] investigated the feasibility of a multimodal e-mental health treatment for depressed patients in a pilot study, and suggested that joint effects of Internet and telephone administered support for depression are promising. In order to further increase compliance, we therefore plan to use telephone support in the form of text-messages in addition to the Internet-based intervention. However, whether extra text-messages would enhance compliance rates for Internet-based PST is not known.

The aims of our RCT will therefore be multiple. First, we will examine the effectiveness of an Internet-based PST self-help intervention on the primary outcome measure depressive symptoms in MS patients and on secondary outcome measures related to depression and MS such as quality of life, fatigue and cognitive functioning. If the intervention shows to be effective, predictors of a favorable outcome will be further explored. Finally, we will investigate whether text-messages added to the intervention will be effective to increase compliance rate of Internet-based treatment.

## Methods

### Study design

The presented study is a RCT in which a 5-week Internet-based self-help PST intervention will be tested versus a waiting list control group. Eligible and consenting patients will be assessed at baseline (T0), within a week after the intervention (T1), at four months (T2) and at ten months follow-up (T3: only the intervention group). The control group will be measured at the same moments in time. Patients in the waiting list condition who still want to participate in the intervention after they completed the four months follow-up assessment are again measured after the intervention (T4), four months later (T5) and at ten months follow-up (T6). Data will be collected by self-report measures administered through the Internet. In addition, a telephone interview at baseline will be carried out by trained research staff.

**Figure 1:**
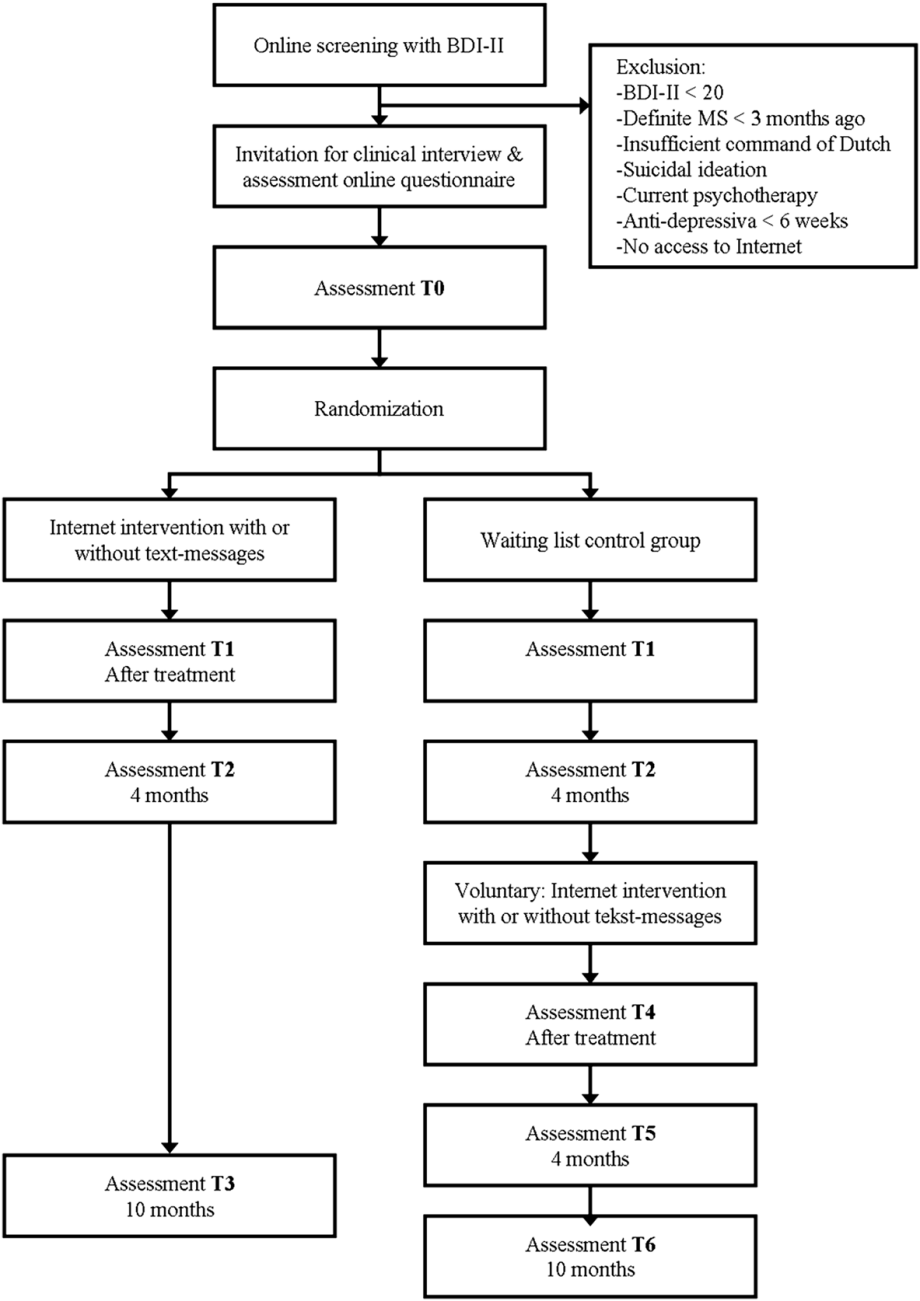
Flowchart of the study design.

Randomization takes place at individual level after baseline measurements (T0). We will use block-randomization with variable block sizes. A randomly allocated number of patients who take part in the intervention will receive supportive text-messages on their mobile phones in addition to the intervention. Allocation will be unknown to the investigators and will be performed by an independent researcher using a computerized random digit generator. The study protocol was approved by the Medical Ethics Committee of the VU University Medical Center (VUmc) (registration number 11/047). Written informed consent is obtained from all participants. Figure 1 displays the flowchart of the study design.

### Recruitment

MS patients will be recruited from the Neurology Department of the VUmc, through calls in MS newsletters and Internet-sites concerning MS (e.g. Dutch MS cooperation; MS Centres), at MS meetings and via MS nurses. Interested patients will be asked to complete an online screening on their depressive symptoms, and several socio-demographic and MS-specific questions (i.e. year of diagnosis, onset of first symptoms, medication usage, type of MS). Eligible patients will be further informed about the study and asked to return written informed consent. Subsequently, patients general practitioner (GP) and neurologist will be informed about participation.

### Inclusion and exclusion criteria

MS patients with depressive symptoms who are willing to take part in an Internet-based self-help course can participate in the study. Patients (i) have to be 18 years or older, (ii) score 20 or higher on the Beck Depression Inventory (BDI-II) [35], indicating a moderate or severe depression and (iii) report a diagnosis of definite MS more than 3 months ago which will also be confirmed by their neurologist. Potential patients will be excluded if they are currently receiving psychotherapy, do not have Internet access, do not have a sufficient command of the Dutch language, or report suicidal ideation. Patients will not be excluded if they are taking prescribed medication for depression or anxiety disorders over 6 weeks with stable dosage.

To exclude patients with suicidal intention, item 9 ‘Suicidal thoughts or wishes’ of the BDI-II will be used. Patients who score ‘3’ (“I would kill myself if I had the chance”), will be contacted, further assessed and excluded and referred to their GP if necessary. Suicidal ideation will be also checked with an interviewer-rated scale of current suicidal ideation of the WHO-Composite International Diagnostic Interview (CIDI, World Health Organiszation (WHO) version 2.1) [36]. Patients with a score of 29 or higher on the BDI-II will be monitored extra.

### Interview

A clinical diagnosis of Major Depression Disorder and/or Anxiety Disorder according to DSM-IV-TR criteria will be established by a standard telephonic interview using the WHO CIDI [36]. Neurological and medical conditions can create difficulties in assessing depression by a structured measurement as the CIDI. The CIDI asks patients how much of their sadness and anhedonia is a result of their medical illness or medications. In case patients indicate that their depressive symptoms are due to these attributes, a psychiatric diagnosis is not given. We decided that in case MS patients reported their MS or MS medication to be the reason for their sadness or anhedonia, the interviewer makes a note and scores the item negatively.

Further, the Perceived Need of Care Questionnaire (PNCQ) [37] will be used to measure patients' utilization of health care resources, needs for mental health care and the meeting of those needs. Further, the telephonic version of the Expanded Disability Status Scale [38] will be used to asses physical (MS) functioning and disability level.

### Outcome measures

#### Primary outcome measure

The primary outcome is the change in depressive symptoms defined by a change in the sum score on the Beck Depression Inventory (BDI-II, second edition) [35,39]. This self-report instrument is most often used to measure depression severity in MS patients [3], and has shown to be valid, reliable and appropriate for the MS population [40,41].

#### Secondary outcome measures

Secondary outcomes will include measures of anxiety, fatigue, cognitive functioning, physical and psychological impact of MS, quality of life, problem solving skills, social support, mastery, satisfaction and compliance rate. The measurement scales mentioned below have shown to be reliable, valid and responsive, and are widely used as an outcome measure in MS research and/or research on psychiatric conditions.

Anxiety will be assessed with the Beck Anxiety Inventory [42] and the subscale of the Hospital Anxiety and Depression Scale [43]. We will use the Fatigue Severity Scale (FSS) [44] and Multiple Sclerosis Neuropsychological Questionnaire (MSNQ) [45] to measure fatigue and cognitive functioning, respectively. In addition, we will use the Multiple Sclerosis Impact Scale (MSIS-29) [46] to assess the physical and psychological impact of MS and the EuroQol quality of life measure comprising a five-part questionnaire (EQ-5D) and a visual analogue self-rating scale (EQ-VAS) [47]. Social support will be measured with the Social Support Inventory [48] which contains questions on details about social support from the four most intimate persons. We will use the Social Problem Solving Inventory-Revised (SPSI-R) to determine individual problem-solving skills [49]. Furthermore, Mastery or locus of control, is measured by an abbreviated version of the Pearlin Mastery Scale [50]. Between follow-ups; psychotherapy and -medication usage will be registered.

With the Client Satisfaction Questionnaire (CSQ-8) [51] we will check whether patients were satisfied with the care they received. The Visual Analogue Scale (VAS) will be assessed to evaluate patients opinions about the intervention as a whole, the website and received support, (via the website and by text-messages if applicable) and additionally explore reasons for drop-out. Feasibility of additional text-messages will further be assessed by compliance rate; data on number of treatment sessions and drop-out will be collected. Table 1 describes the measures used at each assessment point.

**Table 1:** Summary of measures

**Measure**	**T0: Baseline**	**T1: posttreatment**	**T2: 4-months Follow-up**	**T3: 10-months Follow-up**
**Interview:**				
Composite International Diagnostic Interview (CIDI)	X			
Perceived Need of Care Questionnaire (PNCQ)	X			
Expanded Disability Status Scale (EDSS)	X			
**Self-report measurements:**				
Demografics & Medical MS data	X			
Medication and treatment information	X	X	X	X
Beck Depression Inventory (BDI-II)	X	X	X	X
Beck Anxiety Inventory (BAI)	X	X	X	X
Hospital Anxiety and Depression Scale (HADS-A)	X	X	X	X
Fatigue Severity Scale (FSS)	X	X	X	X
Multiple Sclerosis Neuropsychological Questionnaire (MSNQ)	X	X	X	X
Multiple Sclerosis Impact Scale (MSIS-29)	X	X	X	X
EuroQol (EQ-6D)	X	X	X	X
Social Support Inventory	X	X	X	X
Social Problem Solving Inventroy-Revised (SPSI-R)	X	X	X	X
Pearlin Mastery Scale	X	X	X	X
Client Staisfaction Questionnaire (CSQ-8)		X		X
Visual Analoge Scale (VAS) to evaluate the intervention		X		

### Internet-based problem solving treatment

The Internet-based self-help intervention examined in this study is an online problem solving therapy (PST) [16]. We adjusted the original online intervention [28] for MS patients with co-morbid depression, conserving the intent of the PST-based intervention. Modifications concerned additional information about MS and its psychosocial consequences and texts and examples applying to MS patients. We also added a mood-chart to the intervention for daily mood-registration during the course. The mood-chart could stimulate patients to visit the website regularly and therefore enhance compliance. In addition, it offers the possibility to monitor the patients mood. The whole intervention exists of five modules with text, exercises and figures, and is called ‘Worry Less’ (“Minder Zorgen”). Patients are asked to attend one module a week and work on their assignments for at least 2 hours per week.

Patients can access the intervention from their personal computers via the Internet (https://minderzorgen.e-behandeling.nl). Support during the intervention will be provided by trained psychologists and supervised clinical psychology Master students, and consists of communication through brief, weekly e-mails sent through the website and a weekly standardized e-mail to announce a new module. The e-mail correspondence is merely intended to facilitate the patient’s effective use of the self-help method. Patients have the possibility to contact their coach at any moment for additional support via the website.

### Text-messages

As part of the intervention, a number of patients will receive weekly text-messages on their mobile phones to support them during the intervention with the aim to enhance compliance. Patients will be randomly assigned to the intervention with- or without text-messages. In the latest case, patients will receive four standard text-messages a week. Text-messages will be sent to their mobile phones 1) at the start of a new module, 2) during the week to remind patients to fill in the exercise and mood-chart, 3) when homework is handed in by email, and 4) when feedback is returned by their coach. When homework is not handed in on time, patients will receive a reminder text-message. Patients cannot reply to the text-messages.

### Waiting list control group

Patients randomized to the waiting list control group receive no Internet-based PST. In accordance with the procedure of patients who participate in the intervention, patients in the waiting list control group are free to accept any medical or psychological intervention given in the time period of the study. The received mental healthcare will be registered. After completion of the four months follow-up assessment, patients are offered to participate in the Internet-based intervention on voluntary basis irrespective their BDI-II score at T2. Patients who subsequently take part in the intervention and follow-up measurements will be additionally analysed.

### Sample size

The power calculation is based at the comparison at T1 to T0 between the two groups. We want to be able to demonstrate moderate effects (*d* = 0.5) on the primary outcome measure, while using a power 0.80, with alpha set at .05 (two-tailed). Therefore, a total set of N = 64 completers is needed in each condition. Taking into account the drop-out percentage of our pilot, we aim to include 166 patients.

### Analysis

Non-parametric and parametric statistical tests are used to assess differences between the conditions with regard to baseline assessment of all relevant demographic and clinical variables. Missing data will be processed using regression imputation or a conservative Last Observation Carried Forward method (LOCF) (depending on the sort and size of the missing data). Analysis will be based on the intention-to-treat principle. Paired t-tests are used to assess the changes within each condition between pre-treatment and post-treatment. Difference in outcome between the Internet-based intervention and waiting list control group is evaluated by means of mixed model analysis of covariance and baseline differences will be included as a covariate in the analyses. Treatment effect over time will be tested by adding a group*time interaction term into the model. Cohen’s formula will be used to evaluate the magnitude of the effect of the intervention on outcome measures [52]. To calculate clinically significant improvement and recovery we will use the standardized method of Jacobson and Truax [53]. If the intervention shows to be effective, predictors of outcome will be explored by analyses of interaction between patients characteristics and treatment. Data of waiting list patients who decided to participate in the intervention will be analysed separately. Finally, descriptive statistics of compliance rate and satisfaction will be used to explore the feasibility of text-messages as a way to increase compliance rate to Internet-based treatment.

## Discussion

The described study protocol is designed to investigate the effectiveness of an Internet-based self-help PST intervention for depressive symptoms in MS patients in a RCT. Treatment through Internet offers the possibility to reach a group of underserved MS patients who could experience disease-related barriers to participate in face-to-face counselling. Besides, the self-help aspect of Internet-based treatment could be appealing to a group of patients that already is dependent of professionals in the medical circuit because of their MS. According to our knowledge, the effectiveness of Internet-based treatment for depressive symptoms in MS patients has not been examined yet. Our trial will also shed more light on the applicability of PST in itself for patients who suffer from MS.

In order to conduct a high-quality trial, a multidisciplinary team with various expertises related to MS and depression was involved in the development of the intervention and the study design. Ways to prevent early termination during the intervention were carefully taken into account since high drop-out percentages in e-health interventions are a common phenomenon. Although multimodal e-mental health treatment seems a promising candidate to increase compliance, additional research on this subject is needed [34]. Evaluation of the effectiveness of text-messages in addition to Internet-based treatment seems therefore a valuable aspect of our trial. Nevertheless, it is still unclear whether effectiveness of additional text-messages can be determined as the trial is powered to demonstrate effects of the intervention and not of text-messaging. Further, follow-up assessments to explore Internet-based PST effects on the long-term could be considered another strength of our trial since data on enduring treatment effects for depression in MS are often not available [11].

The external validity of our study is high because of the low level of exclusion criteria and the use of Internet that enables us to reach and treat a large part of the MS population. It should be noted that Internet-based treatment may not be suitable for MS patients who are not familiar with the Internet, or who suffer from MS related problems such as impaired vision and arm/hand dysfunction. In addition, we will exclude patients with mild depressive complaints (BDI-II < 20). As a consequence, evidence will be lacking whether the intervention could be effective for this group of patients. However, our pilot-study [29] showed that patient with more severe depressive symptoms experienced more benefit from the intervention than patients with fewer depressive complaints. Moreover, patients with fewer depressive symptoms at baseline (<20) were more likely to drop out of treatment and indicated to be less satisfied with the intervention. A meta-analysis [23] supports this finding and suggests that patients with less severe psychological difficulties may be less motivated to receive ongoing help. Driessen and colleagues concluded in their meta-analysis [54] that psychological treatment might even be more efficacious for more severely depressed patients than low severely depressed patients. For these reasons we decided to focus on MS patients with moderate or high depressive complaints.

To conclude, a study protocol to investigate the effectiveness of Internet-based PST for depressive symptoms in MS patients is presented in order to enhance methodological clarity and further research on treatment for depressed MS patients. Internet-based PST offers the possibility to reach and treat many MS patients with depressive symptoms and to improve the quality of care. Our RCT is aimed at contributing to better recognition and adequate treatment of depressive symptoms in MS patients in the future.

## Competing interests

There are no conflicts of interests for all authors.

## Authors’ contributions

PvO, JD, BU, AB, PC, CP, EC and RB contributed to the design of the study. The study is being coordinated by PvO, JD and BU. The present manuscript was drafted by RB, PvO, JD and BU. All authors contributed to critical revision of the manuscript for important intellectual content. All authors read and approved the final manuscript.

## **Funding**

This work was supported by the Stichting MS Research [grant number 09–678 MS].

## Pre-publication history

The pre-publication history for this paper can be accessed here:

http://www.biomedcentral.com/1471-244X/12/137/prepub
